# Synthetic Curcuminoids Combined with Blue-Light-Mediated Photodynamic Therapy for Anti-Inflammatory Effect and Potential Mechanism on Imiquimod-Induced Psoriasis-like Proliferation of Keratinocytes

**DOI:** 10.3390/ijms27156716

**Published:** 2026-07-27

**Authors:** Lee-Fong Lin, Yu-Chang Chu, Wei-Chun Chen, Jing-Jia Hsu, Ming-Lun Chou, Anren Hu, Sung-Jen Hung

**Affiliations:** 1 Department of Biomedical Sciences and Engineering, Tzu Chi University, Hualien 97004, Taiwan; leelin@gms.tcu.edu.tw (L.-F.L.); 110726106@gms.tcu.edu.tw (Y.-C.C.); 110711151@gms.tcu.edu.tw (W.-C.C.); 110711114@gms.tcu.edu.tw (J.-J.H.); michou1015@gms.tcu.edu.tw (M.-L.C.); 2 Department of Life Sciences, Tzu Chi University, Hualien 97004, Taiwan; 3 Department of Laboratory Medicine and Biotechnology, Tzu Chi University, Hualien 97004, Taiwan; anren@gms.tcu.edu.tw; 4 Department of Dermatology, School of Medicine, Tzu Chi University, Hualien 97004, Taiwan; 5 Department of Dermatology, Hualien Tzu Chi Hospital, Buddhist Tzu Chi Medical Foundation, Hualien 97002, Taiwan

**Keywords:** curcumin analog, photodynamic therapy, psoriasis, blue light, anti-inflammation

## Abstract

Psoriasis is characterized by the rapid proliferation of keratinocytes, leading to erythematous plaques with silvery scales, skin pain, and impaired quality of life. Photodynamic therapy (PDT) has emerged as a promising treatment modality for various skin disorders, including psoriasis. Curcumin, a natural photosensitizer, possesses potent anti-inflammatory and antioxidant properties. In this study, we investigated the anti-inflammatory potential of synthetic curcuminoids combined with blue-light-mediated PDT for psoriasis treatment using human keratinocyte (HaCaT) cells. MTT assays demonstrated that the synthetic curcuminoids (1E,6E)-1,7-bis(5-methylthiophen-2-yl)hepta-1,6-diene-3,5-dione (No. 11) and (1E,6E)-1,7-di(thiophen-2-yl)hepta-1,6-diene-3,5-dione (No. 12) exhibited minimal cytotoxicity toward HaCaT cells. Upon ultraviolet–visible light irradiation, curcuminoid-treated cells generated intracellular reactive oxygen species, confirming the photodynamic activity induced by blue light exposure. Treatment with synthetic curcuminoids significantly suppressed the secretion of pro-inflammatory cytokines IL-17A/F and IL-8 in imiquimod-stimulated HaCaT cells, a commonly used psoriasis-like inflammatory model, indicating notable anti-inflammatory effects. Western blot analysis further revealed an increased Bax/Bcl-2 ratio in cells treated with synthetic curcuminoids No. 11 or No. 12 under photodynamic irradiation, suggesting the induction of apoptosis. Synthetic curcuminoids combined with blue-light PDT may represent a promising and cost-effective strategy for psoriasis.

## 1. Introduction

Psoriasis is a chronic immune-mediated inflammatory skin disorder with a strong genetic predisposition and complex pathophysiology involving both innate and adaptive immune responses [1,2,3]. The disease is characterized by erythematous plaques, scaling, and epidermal hyperplasia, which significantly affect patients’ quality of life. Although psoriasis primarily manifests in the epidermis, particularly in keratinocytes, its pathogenesis involves intricate interactions between keratinocytes and multiple immune cell populations in the dermis. Disease progression generally consists of an initiation phase triggered by environmental factors such as pathogens, trauma, or medications [4], followed by a chronic maintenance phase sustained by persistent inflammatory signaling [5]. Despite extensive research, the precise contributions of keratinocytes and immune cells to psoriasis development remain incompletely understood.

Keratinocytes are now recognized as active participants in psoriasis pathogenesis rather than passive targets of inflammation. In psoriatic lesions, keratinocytes undergo abnormal proliferation and differentiation while producing excessive antimicrobial peptides and inflammatory mediators associated with innate immunity [5,6]. Crosstalk between keratinocytes and immune cells amplifies inflammatory cascades and contributes to the characteristic clinical manifestations of psoriasis. In particular, epidermal keratinocytes secrete chemokines such as chemokine (C-X-C motif) ligand 1 (CXCL1) and interleukin-8 (IL-8/CXCL8), which recruit and activate immune cells involved in both innate and adaptive immune responses.

Among the inflammatory pathways implicated in psoriasis, the interleukin-17 (IL-17) signaling axis has emerged as a critical regulator of disease initiation, progression, and maintenance [7,8,9]. Several IL-17 family cytokines, including IL-17A, IL-17F, IL-17C, IL-17E, IL-17B, and IL-17D, have been identified, although the complete receptor complexes and signaling mechanisms remain incompletely elucidated [10]. Elevated expression levels of IL-17A, IL-17A/F, and IL-17C have been detected in psoriatic skin and are associated with increased expression of inflammatory mediators such as CXCL1, CXCL8, and DEFB4 in keratinocyte stem cells and early transit amplifying cells [10]. Furthermore, IL-17A and IL-17A/F contribute significantly to keratinocyte proliferation, abnormal differentiation, and the expression of psoriasis-related markers.

A hallmark of psoriasis is dysregulated epidermal homeostasis, characterized by excessive keratinocyte proliferation, impaired differentiation, and reduced apoptosis [10]. Teige et al. [11] suggested that abnormal keratinocyte differentiation and proliferation contribute to chronic skin inflammation and persistent immune activation. Previous studies have indicated that the psoriatic epidermis may result from defects within the transit-amplifying (TA) cell compartment rather than from alterations in cell cycle duration alone [12,13]. Moreover, Weatherhead et al. [14] demonstrated that ultraviolet B irradiation induced apoptosis more readily in TA cells than in keratinocyte stem cells, highlighting differential apoptotic sensitivity among epidermal cell populations. Notably, psoriatic TA cells exhibit significantly lower apoptotic rates than normal TA cells [10,14], suggesting that therapies that induce apoptosis in hyperproliferative keratinocytes may provide therapeutic benefit for psoriasis patients.

Photodynamic therapy (PDT) has gained considerable attention as an effective treatment modality for various cancers and cutaneous infectious diseases [15,16,17]. PDT relies on the interaction of three essential components: a photosensitizer (PS), a specific wavelength light source, and molecular oxygen. Upon light activation, the PS generates singlet oxygen (^1^O_2_) and other reactive oxygen species (ROS), leading to oxidative damage, apoptosis, and immune modulation [18,19]. In dermatology, PDT has shown promising therapeutic potential not only for malignancies but also for inflammatory skin disorders, including psoriasis [20]. Given the growing concern about antibiotic resistance and the limitations of current systemic therapies [21], PDT-based approaches may offer alternative, cost-effective treatment strategies with fewer systemic side effects.

Curcumin, a natural polyphenolic compound isolated from the rhizomes of *Curcuma longa* (turmeric), has long been recognized for its anti-inflammatory, antioxidant, and therapeutic properties [22,23,24,25]. In addition to curcumin, *Curcuma longa* contains other related compounds, including demethoxycurcumin and bisdemethoxycurcumin, collectively referred to as curcuminoids [26]. Importantly, curcumin functions as a natural PS, making it a promising candidate for PDT applications [25,27]. Previous studies demonstrated that synthetic curcuminoids No. 11 and No. 12 exhibit antibacterial activity when combined with PDT [28]. However, their potential immunomodulatory effects on psoriasis-related inflammation and keratinocyte dysfunction remain largely unexplored.

In the present study, we investigated the anti-inflammatory potential of synthetic curcuminoids No. 11 and No. 12, combined with blue-light-mediated PDT, in imiquimod-induced psoriasis like proliferation of human keratinocyte (HaCaT) cells.

## 2. Results

### 2.1. Absorption Light Spectrums of Synthetic Curcuminoids No. 11 and No. 12 Confirm Their Functions as PS by UV–Vis Studies

**Figure 1:**
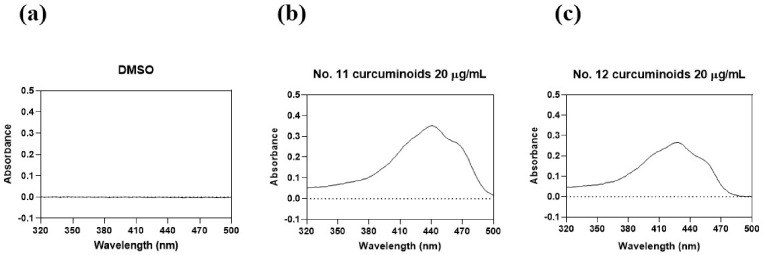
Absorption light spectrum of curcuminoids No. 11 and No. 12, respectively, by UV-Vis assays. (**a**) DMSO was used as a negative control, eliciting no absorbance within the blue light region (400–500 nm). (**b**) Absorption light spectrum of curcuminoids No. 11 showed a specific absorption peak within the blue light region with a λmax of 441 nm. (**c**) Absorption light spectrum of curcuminoids No. 12 indicated an absorption peak within the blue light region with a λmax of 427 nm.

To investigate whether synthetic curcuminoids No. 11 and No. 12 can act as PSs similar to natural curcumin, their light absorption properties were analyzed using a NanoDrop spectrophotometer. Solutions with DMSO of No. 11 or No. 12 (20 µg/mL) were exposed to continuous UV–Vis light spanning 190–840 nm, and their relative absorbance spectra were recorded. DMSO served as a negative control and exhibited negligible absorbance across the blue-light region (400–500 nm), confirming minimal background interference (Figure 1a). In contrast, both synthetic curcuminoids demonstrated distinct absorption peaks within the blue-light range. The maximum absorption wavelengths (λmax) were identified at 441 nm for No. 11 and 427 nm for No. 12 (Figure 1b,c). These values are consistent with previously reported spectra for these compounds, which show λmax values of approximately 440 nm and 426 nm, respectively [28]. Overall, these results confirm that synthetic curcuminoids No. 11 and No. 12 exhibit strong absorption in the blue-light region, supporting their ability to function as PSs. This characteristic is essential for their potential application in PDT, as it is for natural curcumin.

### 2.2. Determination of Maximal Cell Viability Concentrations of Synthetic Curcuminoids No. 11 and No. 12 for Their Use in Subsequent HaCaT Studies

**Figure 2:**
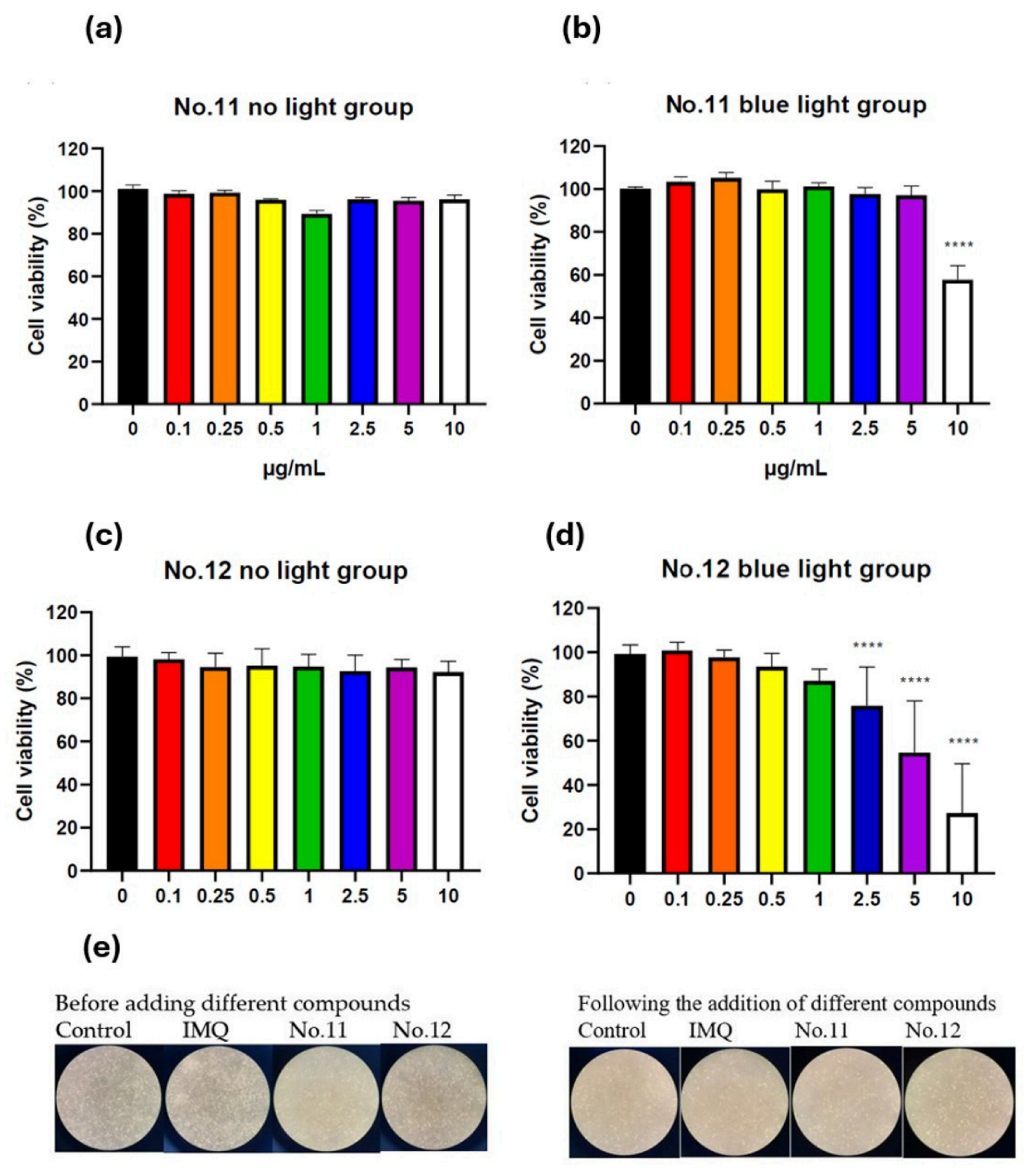
MTT assays determining the maximal cell viability concentrations of synthetic curcuminoids No. 11 and No. 12 in HaCaT cells. (**a**) Concentrations of 0.1–10 µg/mL of curcuminoid No. 11 were used to treat HaCaT cells under no light conditions. Concentrations of 0 represent media with DMSO as the negative control. Cell viability (%) was determined by using the MTT assay as described in Section 4 (n = 15). (**b**) Same concentrations of 0.1–10 µg/mL of curcuminoid No. 11 were used to treat HaCaT cells under 1 min blue light irradiation. Cell viability (%) was determined similarly by using the MTT assay (n = 15). (**c**) A Similar MTT study was performed as that of (**a**), except No. 11 was replaced by No. 12 (n = 15). (**d**) A similar assay was conducted as that in (**b**); however, No. 12 instead of No. 11 was employed. Cell viability (%) was measured to determine the maximal cell viability concentration of No. 12 (n = 15). (**e**) Cell graphs of keratinocytes (HaCaT) before and after different treatments were shown. A one-way ANOVA assay was used in this study. The average was shown as ±SEM. **** *p* < 0.0001.

Hung et al. [28] previously demonstrated that synthetic curcuminoids No. 11 and No. 12 exhibit effective photodynamic antibacterial activity against Gram-positive aerobic bacteria, including *Staphylococcus aureus* and *Staphylococcus epidermidis*. However, their potential cytotoxicity toward human keratinocytes (HaCaT cells) has not been evaluated. In the present study, the cytotoxicity of No. 11 and No. 12 was first assessed in HaCaT cells at concentrations ranging from 0.1 to 10 µg/mL, both in the absence and presence of 1 min blue-light irradiation, using the MTT assay. The results showed that, in the absence of light exposure, both curcuminoids were non-cytotoxic across the tested concentration range (Figure 2a,c). In contrast, photodynamic activation induced concentration-dependent cytotoxicity. Specifically, 10 µg/mL of No. 11 significantly reduced cell viability under blue-light irradiation, whereas No. 12 exhibited significant cytotoxicity at 2.5 µg/mL and above under the same conditions (Figure 2b,d).

Based on these findings, concentrations of 5 µg/mL for No. 11 and 1 µg/mL for No. 12, representing the highest non-cytotoxic doses under experimental conditions—were selected for subsequent experiments. In addition, blue light irradiation alone did not induce measurable cytotoxicity, confirming that phototoxic effects were dependent on the presence of the curcuminoids (Figure 2b,d, 0 µg/mL control). Furthermore, no cytotoxicity was observed in HaCaT cells treated with IMQ, 5 µg/mL No. 11, or 1 µg/mL No. 12 under the conditions used (Figure 2e).

### 2.3. ROS Produced Following Synthetic Curcuminoids No. 11 or No. 12 and t-BuOOH Treatment Are Generated Inside HaCaT Cells

**Figure 3:**
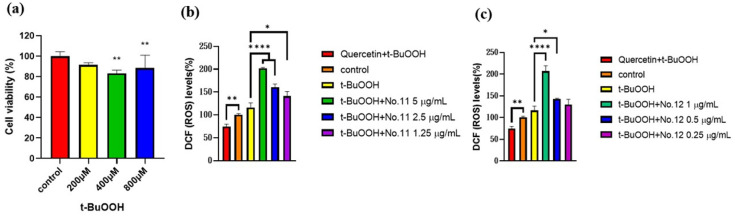
Intracellular ROS Production in HaCaT cells after curcuminoids No. 11 or No. 12 in the presence of t-BuOOH and under blue light exposure. (**a**) Cell viability assay determining the concentration of t-BuOOH used as an oxidant in this study (n = 5). (**b**,**c**). Intracellular ROS formation was detected using the method already described. Different colors represent HaCaT cells that were exposed to distinct concentrations of curcuminoids No. 11 or No. 12 with oxidant t-BuOOH under blue light detection (n = 3). Quercetin was employed as a positive control (antioxidant), shown in red. One-way ANOVA and *t*-test were used, and the average was indicated as ±SEM. * *p* < 0.05; ** *p* < 0.01; **** *p* < 0.0001.

According to a previous report [28], the antibacterial activity of synthetic curcuminoids No. 11 and No. 12 under blue-light irradiation was primarily attributed to the generation of ROS, leading to apoptosis-mediated elimination of Gram-positive bacteria, including *Staphylococcus aureus* and *Staphylococcus epidermidis*. However, that study did not determine whether ROS were produced intracellularly or extracellularly, nor did it examine how ROS contributed to cell death following curcuminoid exposure. To further clarify the subcellular origin of ROS generation, relevant literature was reviewed [20,29], and an intracellular ROS assay was performed as described in Section 4. This assay is based on the conversion of non-fluorescent DCFH-DA into fluorescent DCF following deacetylation by intracellular esterases. The resulting fluorescence intensity reflects intracellular ROS levels after DCFH oxidation by ROS. t-BuOOH was used as an exogenous oxidative stress inducer, as previously reported [30], to establish a ROS-generating model in HaCaT cells. To determine a non-cytotoxic working concentration, HaCaT cells were exposed to different concentrations of t-BuOOH and assessed using the MTT assay. The results showed that 200 µM t-BuOOH did not significantly affect cell viability, whereas 400 µM and 800 µM induced marked cytotoxicity (Figure 3a). Therefore, 200 µM t-BuOOH was selected for subsequent ROS experiments.

The intracellular ROS assay demonstrated that treatment with synthetic curcuminoids No. 11 or No. 12 significantly increased ROS levels in a dose-dependent manner in the presence of 200 µM t-BuOOH (Figure 3b,c). These findings indicate that intracellular ROS production is positively correlated with curcuminoid concentration. This is particularly important, as ROS generation is a key component of PDT. Quercetin (156 µg/mL) was used as a positive antioxidant control. Collectively, these results confirm that synthetic curcuminoids No. 11 and No. 12 can penetrate HaCaT cells and promote intracellular ROS formation under blue light, supporting their potential application as PSs in PDT.

### 2.4. Synthetic Curcuminoids No. 11 and No. 12 Possess Anti-Inflammatory Effects on IMQ-Treated HaCaT Cells Under Blue Light Exposure

Curcumin has been widely reported to exert anti-inflammatory effects by suppressing the production of multiple pro-inflammatory cytokines [31]. To investigate whether synthetic curcuminoids No. 11 and No. 12 share similar properties, culture supernatants were collected from HaCaT cells treated with either curcuminoid No. 11 or No. 12 in the presence of IMQ, with or without blue-light irradiation. IMQ is known to induce psoriasis-like inflammatory responses in keratinocytes [32,33]. As previously reported by Varma et al. [32], exposure of HaCaT cells to 100 µM IMQ for up to 24 h results in pronounced cytotoxicity, whereas a 1 h treatment induces minimal cell damage. Therefore, 100 µM IMQ for 1 h was used in the present study to establish an inflammatory model. The present findings confirmed that synthetic curcuminoids No. 11 and No. 12 act as PSs and that ROS production was significantly enhanced under blue-light irradiation (Figure 1b,c and Figure 3b,c). These observations satisfy the essential requirements for PDT, namely a PS, light exposure, and molecular oxygen. Given the central role of IL-17 signaling in psoriasis pathogenesis, we further examined whether synthetic curcuminoids could modulate inflammatory cytokine release. HaCaT cells were pretreated with 100 µM IMQ for 1 h to induce a psoriasis-like inflammatory state, followed by exposure to curcuminoids No. 11 or No. 12 with or without blue-light irradiation. Based on previous studies demonstrating an association between IL-17A/F expression and psoriatic epidermal inflammation [7,8,9], IL-17A/F levels in culture supernatants were first quantified by ELISA.

**Figure 4:**
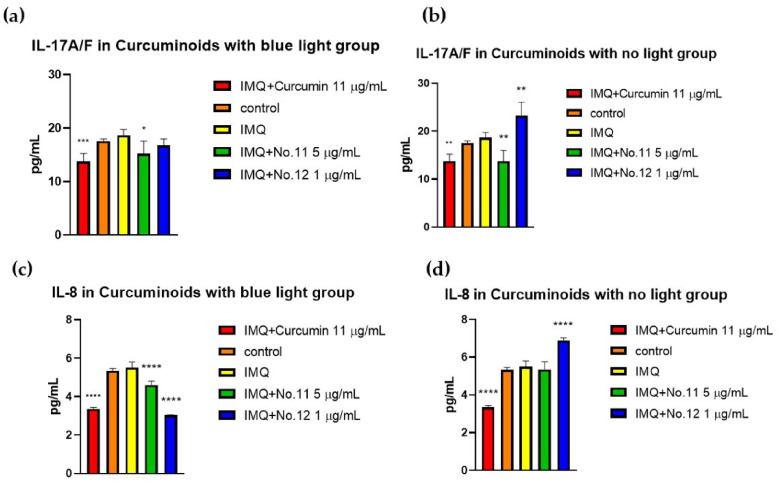
Quantitative analysis of human cytokines IL-17A/F and IL-8 in IMQ-treated HaCaT cells following processing with synthetic curcuminoids No. 11 or No. 12 with/without blue light exposure. (**a**) Illustrates the effect of curcuminoids No. 11/12, plus the presence of IMQ, on the production of IL-17A/F under 1 min blue light irradiation (n = 4). (**b**) Under no blue light exposure, IL-17A/F amount was detected after curcuminoids No. 11 and No. 12 plus IMQ treatment, respectively (n = 4). (**c**) IL-8 quantities were determined in response to curcuminoids No. 11 or No. 12 plus blue light irradiation for IMQ-treated HaCaT cells (n = 4). (**d**) Without blue light exposure, IL-8 level was detected for different curcuminoids and IMQ-processed HaCaT cells (n = 4). One-way ANOVA and *t*-test were used for statistical analysis. The average was shown as ±SEM. * *p* < 0.05; ** *p* < 0.01; *** *p* < 0.001; **** *p* < 0.0001.

As shown in Figure 4a,b, IMQ-induced secretion of IL-17A/F was significantly reduced in cells treated with curcuminoid No. 11, reaching levels comparable to those observed with curcumin (positive control), regardless of light exposure. These results suggest that No. 11 may suppress psoriasis-related inflammatory responses, potentially through modulation of intracellular signaling pathways independent of photodynamic activation. In contrast, No. 12 produced only a modest and non-significant reduction in IL-17A/F levels compared with curcumin (Figure 4a). Furthermore, IL-8, a key pro-inflammatory chemokine, was markedly reduced to levels similar to those of curcumin, but only in the groups receiving both curcuminoid treatment and blue-light irradiation. This indicates that the anti-inflammatory effects on IL-8 production are dependent on photodynamic activation. Overall, these findings demonstrate that synthetic curcuminoids No. 11 and No. 12 exert anti-inflammatory effects in IMQ-stimulated HaCaT cells, with differential contributions from light-dependent and light-independent mechanisms.

### 2.5. Anti-Inflammatory Effect on IL-8 mRNA Production in HaCaT Cells Treated with Synthetic Curcuminoids No. 11 and No. 12

**Figure 5:**
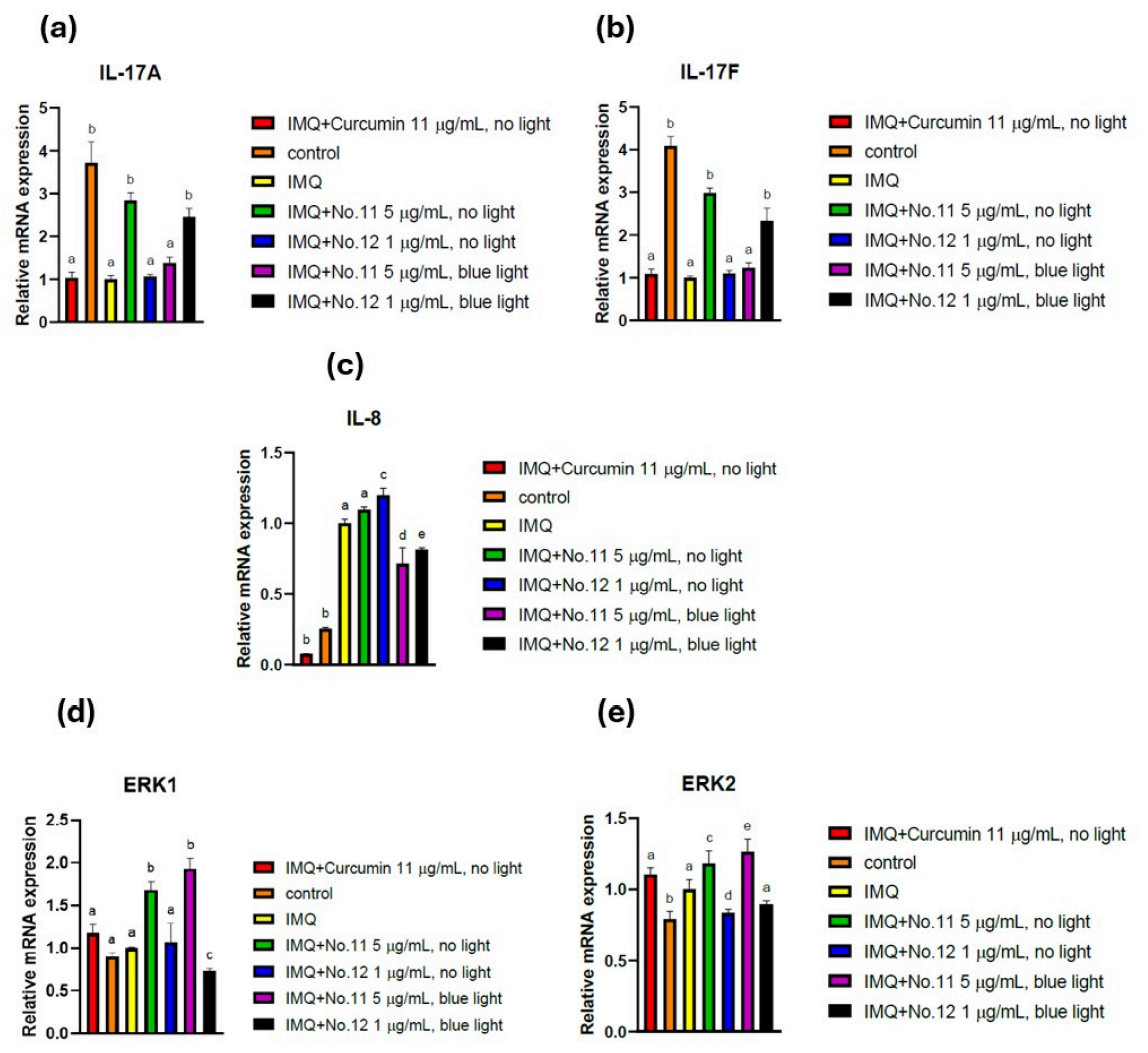
Different cytokines and ERK mRNA analysis by qRT-PCR studies on HaCaT cells with distinct treatments. (**a**,**b**) Relative IL-17A and IL-17F mRNA expression levels on HaCaT cells with/without IMQ plus curcuminoids No. 11 and No. 12, respectively, in the presence/absence of blue light exposure. Curcumin was used as a positive control. (**c**) IL-8 mRNA quantities were determined on HaCaT cells following different treatments. (**d**,**e**) Relative quantitative analysis of ERK1 and ERK2 mRNAs with treatments shown in different colors. One-way ANOVA studies were performed. The average is elicited as ±SEM. The same letter shown in the figures indicates no substantial difference, while the altered letter elicits a statistically significant change.

This study demonstrated that synthetic curcuminoids No. 11 and No. 12 exert anti-inflammatory effects on IMQ-stimulated HaCaT cells under blue-light irradiation (Figure 4c,d). To determine whether these effects occur at the transcriptional or post-transcriptional level, qRT-PCR analysis was performed. The results showed that IL-17A and IL-17F mRNA expression in HaCaT cells treated with IMQ in combination with curcuminoid No. 11 was restored to levels comparable to the negative control, regardless of blue-light exposure (Figure 5a,b). These findings indicate that the reduction in IL-17A/F protein levels observed with No. 11 in the absence of light (Figure 4b) is not mediated at the mRNA level. However, under blue-light irradiation, the decrease in IL-17A/F protein levels induced by No. 11 (Figure 4a) was accompanied by a significant reduction in IL-17A and IL-17F mRNA expression, suggesting transcriptional regulation under photodynamic conditions. In contrast, curcuminoid No. 12 exhibited a distinct regulatory pattern. Although No. 12 produced a slight reduction in IL-17A/F protein levels under both light and dark conditions (Figure 4a), these changes were not associated with corresponding alterations in IL-17A or IL-17F mRNA expression under blue-light exposure. Interestingly, in the absence of light, No. 12 significantly decreased IL-17A and IL-17F mRNA levels compared with the IMQ control, despite the observed increase in protein levels under these conditions (Figure 5a,b).

Regarding IL-8, both mRNA and protein levels induced by IMQ were markedly suppressed by curcuminoids No. 11 and No. 12, but only under blue-light irradiation (Figure 4c,d and Figure 5c). No significant changes were observed in the absence of light. These findings indicate that the inhibitory effects of No. 11 and No. 12 on IL-8 production under photodynamic conditions are regulated at the transcriptional level.

To further explore the underlying mechanisms, we examined the involvement of key inflammatory signaling pathways. Previous studies have shown that activation of the IL-17 receptor complex can stimulate downstream signaling cascades, including extracellular signal-regulated kinase (ERK), protein kinase B (AKT), and nuclear factor-kappa B (NF-κB) p65 pathways, thereby promoting inflammatory responses [34,35]. In the present study, we assessed whether the anti-inflammatory effects of curcuminoids were associated with changes in ERK1 and ERK2 mRNA expression under blue-light irradiation. As shown in Figure 5d,e, ERK1 mRNA expression was significantly reduced in the IMQ plus No. 12 group compared with the IMQ control, whereas ERK2 expression remained largely unchanged.

Overall, these results suggest that synthetic curcuminoids No. 11 and No. 12 modulate inflammatory responses in IMQ-stimulated keratinocytes in a light-dependent manner. However, the involvement of the ERK signaling pathway in mediating these effects requires further investigation.

### 2.6. Western Blotting Analysis Revealed Increased Apoptosis in HaCaT Cells Treated with Synthetic Curcuminoids No. 11 or No. 12 Under Blue Light Irradiation

**Figure 6:**
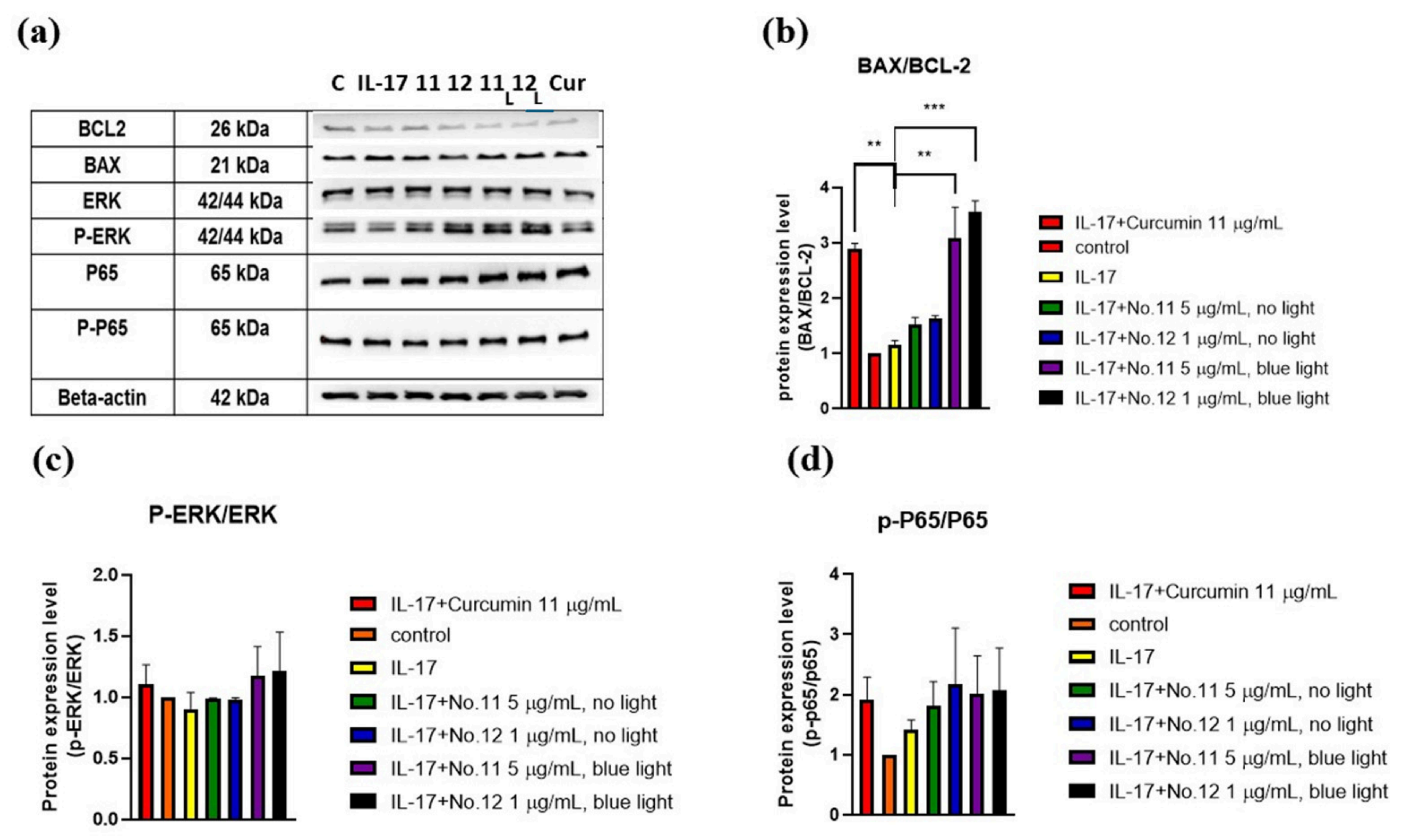
Western blotting analysis to examine apoptosis-related proteins, including potential signaling proteins implicated in the anti-inflammatory effect of synthetic curcuminoids No. 11 and No. 12. (**a**) Western blotting images were captured on HaCaT cells treated with various conditions. C indicates a negative control without any treatments, whereas 11 and 12 represent curcuminoids No. 11 and No. 12 treatments. 11_L_ and 12_L_ mean HaCaT cells were processed with curcuminoids No. 11 or No. 12 in the presence of 1 min blue light exposure. Cur shows curcumin treatment only, which served as a positive control. (**b**) The BAX/BCL2 ratio was calculated based on the Western blotting images shown in (**a**). (**c**,**d**) P-ERK/ERK and P-P65/P65 ratios were calculated similarly to those in (**b**). One-way ANOVA assays were performed. The average was shown as ±SEM. ** *p* < 0.01; *** *p* < 0.001.

Following transcriptional validation (Figure 5), protein expression was further examined in HaCaT cells treated with IL-17 in combination with synthetic curcuminoids No. 11 or No. 12, with or without blue-light irradiation, using Western blot analysis. IL-17 is known to induce psoriasis-like inflammatory responses in keratinocytes, similar to those triggered by IMQ stimulation [36]. The results showed no significant difference in the BAX/BCL2 ratio, an indicator of apoptotic activity, between the IL-17-treated control group and cells treated with IL-17 plus either curcuminoid No. 11 or No. 12 under dark conditions. In contrast, exposure to blue light in combination with IL-17 and either No. 11 or No. 12 markedly increased the BAX/BCL2 ratio. This effect was comparable to that observed with curcumin, the positive control (Figure 6a,b). These findings indicate that synthetic curcuminoids No. 11 and No. 12 induce apoptosis in keratinocytes only when activated by photodynamic treatment. This observation is consistent with the dose-dependent increase in intracellular ROS production shown in Figure 3, suggesting that ROS generation plays a central role in curcuminoid-mediated apoptosis. Given that impaired apoptotic regulation contributes to keratinocyte hyperproliferation and epidermal thickening in psoriasis, these results support the potential therapeutic application of the synthetic curcuminoids No. 11 and No. 12 in PDT for psoriasis.

Because IL-17A is a key inducer of multiple immune-related proteins involved in inflammatory signaling within psoriatic keratinocytes [37], we further investigated whether synthetic curcuminoids affect downstream signaling pathways. ERK and NF-κB p65 are well-established mediators of IL-17A/F-driven inflammatory responses [34,35]. However, Western blot analysis revealed that neither curcuminoid No. 11 nor No. 12 significantly altered the phosphorylation ratios of ERK (p-ERK/ERK) or p65 (p-p65/p65) following IL-17 stimulation, regardless of blue-light irradiation (Figure 6c,d). These findings suggest that the anti-inflammatory and pro-apoptotic effects of synthetic curcuminoids No. 11 and No. 12 are not primarily mediated through modulation of the ERK or NF-κB p65 signaling pathways, indicating that alternative molecular mechanisms may be involved.

## 3. Discussion

Synthetic curcuminoids No. 11 and No. 12 are structural analogs of curcumin, a naturally occurring compound well known for its anti-inflammatory and anticancer activities [25,27,36]. Consistent with the findings of Woźniak et al. [38], who demonstrated that natural curcumin is non-cytotoxic to HaCaT cells at concentrations up to 10 µM (3.65 µg/mL), our results showed that synthetic curcuminoids No. 11 and No. 12 also exhibited minimal cytotoxicity under both dark and blue-light conditions at appropriate concentrations (Figure 2). In addition, these synthetic curcuminoids are relatively simple and inexpensive to produce, suggesting potential economic advantages if comparable anti-inflammatory efficacy can be achieved clinically.

ELISA analysis demonstrated that treatment with synthetic curcuminoids significantly reduced the secretion of IL-17A/F and IL-8 in IMQ-stimulated HaCaT cells, particularly under blue-light irradiation (Figure 4). Among the tested compounds, curcuminoid No. 11 markedly suppressed IL-17A/F production and, consequently, reduced downstream IL-8 secretion under photodynamic conditions. Curcuminoid No. 12 also significantly inhibited IL-8 release, indicating that both synthetic curcuminoids possess considerable anti-inflammatory activity. Previous studies have shown that elevated IL-17 levels contribute to epidermal thickening, abnormal keratinocyte differentiation, pathogenic scaling, and increased IL-8 production in psoriasis [36]. Although natural curcumin has been reported to reduce inflammatory cytokine production in IMQ-induced psoriasis-like mouse models [39], studies examining its effects on IL-17 and IL-8 regulation specifically in the context of PDT remain limited.

Furthermore, Western blot analysis demonstrated that synthetic curcuminoids No. 11 and No. 12 promoted apoptosis in IL-17-stimulated keratinocytes under blue-light irradiation (Figure 6), further supporting their potential utility as PSs in PDT. Collectively, these findings suggest that synthetic curcuminoid-mediated PDT may represent a potential anti-inflammatory and photodynamic regulatory effects of curcuminoid-PDT at the cellular level. Further validation using more physiologically relevant models—such as 3D organotypic skin equivalents or in vivo animal models—will be essential in future studies to confirm these translational benefits.

IL-8 (CXCL8) is a chemokine predominantly produced by activated keratinocytes and innate immune cells in response to upstream cytokines such as IL-17A/F and tumor necrosis factor-α. Its primary biological function is to recruit neutrophils into the epidermis, contributing to the formation of Munro’s microabscesses, a histopathological hallmark of psoriasis [40]. In contrast, IL-17A and IL-17F are mainly secreted by Th17 cells and act as upstream pro-inflammatory cytokines that stimulate keratinocytes to produce additional inflammatory mediators, including IL-6, granulocyte-macrophage colony-stimulating factor, CXCL1, and IL-8, thereby amplifying inflammatory responses and promoting keratinocyte hyperproliferation [41].

In the present study, IMQ stimulation of HaCaT cells induced robust IL-8 production, suggesting that IL-8 may serve as a more sensitive marker of keratinocyte activation than IL-17A/F in this experimental model. Although IL-17A/F are recognized as central drivers of immune dysregulation in psoriasis and are major therapeutic targets of biologic agents, IL-8 primarily reflects downstream chemotactic and inflammatory responses. These mechanistic differences may partially explain the differential modulation of IL-17A/F and IL-8 observed following curcuminoid treatment.

The significant reduction in IL-8 levels following treatment with synthetic curcuminoids No. 11 and No. 12 under blue-light irradiation (Figure 4c), and there seems to be a downward trend of Erk1 mRNA expression at No. 12 under blue-light irradiation. We propose that blue light PDT generates excessive reactive oxygen species (ROS), which suppresses IMQ-induced MAPK signaling, including ERK1, thereby reducing IL-8 production. In addition, Western blotting results revealed that both synthetic curcuminoids No. 11 and No. 12 induced keratinocyte apoptosis following IL-17 stimulation with blue light irradiation (Figure 6b), further suggesting the anti-inflammatory and anti-proliferative potential of curcuminoids in blue light PDT. We further propose that the possible mechanisms include ROS-induced mitochondrial dysfunction, which activates the intrinsic apoptotic pathway via an increased BAX/BCL2 ratio, ultimately promoting caspase-dependent apoptosis of inflamed keratinocytes. Further research is needed to confirm the relevant molecular mechanisms.

Although no corresponding decreases in phosphorylated ERK (p-ERK) or phosphorylated p65 (p-p65) were detected (Figure 6c,d) under the IL-17 stimulation model. IL-8 expression is primarily regulated through canonical NF-κB and mitogen-activated protein kinase (MAPK) pathways, including ERK, c-Jun N-terminal kinase, and p38, in response to inflammatory cytokines such as tumor necrosis factor-α and IL-17A. Similarly, IL-17A/F signaling through the IL-17RA/RC receptor complex and ACT1 adaptor protein activates NF-κB, MAPKs, and C/EBP transcription factors while stabilizing inflammatory mRNAs [42,43]. Additional cross-talk with other pathways, including Janus kinase/signal transducer and activator of transcription and non-canonical NF-κB signaling, may also contribute to psoriasis-associated inflammation [44].

Because p-ERK and p-p65 are classical mediators of inflammatory cytokine transcription, the absence of significant alterations in these signaling molecules suggests that the anti-inflammatory effects of synthetic curcuminoids may involve alternative mechanisms. Possible explanations include modulation of mRNA stability, epigenetic regulation, post-transcriptional control, or involvement of non-canonical signaling pathways. Nevertheless, the present findings indicate that synthetic curcuminoids combined with blue-light PDT can suppress inflammatory activation and keratinocyte hyperproliferation in psoriasis-like conditions, supporting their anti-inflammatory potential for psoriatic skin lesions.

Compared with established PSs such as protoporphyrin IX generated from 5-aminolevulinic acid (5-ALA) or methyl aminolevulinate (MAL), synthetic curcuminoids may offer several advantages. Although 5-ALA- and MAL-mediated PDT activated by 630 nm red light has been widely used for skin cancer treatment, these therapies are often associated with treatment-related pain, prolonged photosensitivity, and strong dependence on oxygen-mediated ROS generation [45,46]. Blue light photodynamic therapy has also been reported in various studies for clinical dermatologic applications, including skin cancers (such as BCC, actinic keratosis, or actinic cheilitis), cutaneous infectious disorders (such as acne), and chronic inflammatory skin diseases (such as psoriasis and rosacea) [47,48]. Psoriatic lesions are relatively superficial and localized primarily within the epidermis and dermal–epidermal junction, allowing efficient penetration of synthetic curcuminoids into keratinocytes while enabling effective activation by blue light. Moreover, unlike 5-ALA or MAL, synthetic curcuminoids may possess intrinsic anti-inflammatory properties and potentially exhibit fewer long-term adverse photosensitive effects. So, these characteristics suggest that synthetic curcuminoids No. 11 and No. 12 could be promising candidates for photodynamic agents, particularly for inflammatory dermatological diseases such as psoriasis.

## 4. Materials and Methods

### 4.1. Chemical Structures of Curcuminoids, Including Curcuminoids No. 11 and No. 12

(1*E*,6*E*)-1,7-Bis(5-methylthiophen-2-yl)hepta-1,6-diene-3,5-dione (No. 11):

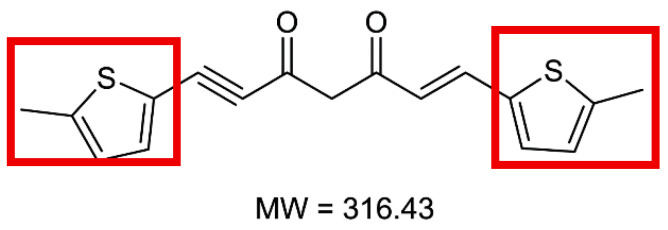



(1*E*,6*E*)-1,7-di(thiophen-2-yl)hepta-1,6-diene-3,5-dione (No. 12):

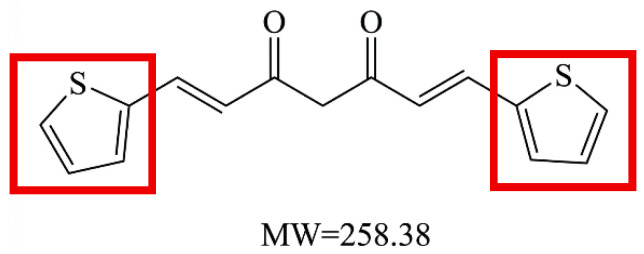



Synthetic compounds No. 11 and No. 12 are thiophene-containing curcuminoid analogs designed to potentially improve photophysical properties and photodynamic performance, as reported previously [49,50]. These structural modifications were introduced to improve the therapeutic potential and to highlight the novelty and design rationale of the curcuminoid derivatives. Synthetic curcuminoids No. 11 and No. 12 were synthesized as previously described [28] and were kindly provided by Dr. Tzenge-Lien Shih from the Department of Chemistry, Tamkang University, Taiwan. The remaining compounds were stored at −20 °C until further use.

### 4.2. HaCaT Cell Culture

HaCaT cells were cultured in Dulbecco’s modified Eagle’s medium supplemented with 0.1 mM glutamine, 25 mM HEPES, 10% fetal bovine serum, 0.36 g sodium bicarbonate, and 1% penicillin–streptomycin (Gibco, Carlsbad, CA, USA). Cells were maintained in a humidified incubator at 37 °C with 5% CO_2_. For subculture, cells were detached using trypsin–EDTA and counted using a hemocytometer with trypan blue exclusion under a light microscope. The cell concentration was adjusted to 2 × 10^5^ cells/mL, and appropriate numbers of cells were seeded into culture plates of different sizes for subsequent experiments. HaCaT cells were kindly provided by Dr. Norbert E. Fusenig (Institute of Biochemistry, German Cancer Research Center, Heidelberg, Germany).

### 4.3. Ultraviolet–Visible (UV-Vis) Molecular Absorption Spectroscopy

UV–Vis light within a wavelength range of 190–840 nm was used to evaluate the light absorption properties of synthetic curcuminoids No. 11 and No. 12. Both compounds were prepared at a concentration of 20 µg/mL with DMSO, as previously described by Hung et al. [28], and continuously exposed to light irradiation. Absorption spectra were recorded using a NanoDrop spectrophotometer (Thermo Fisher Scientific, Waltham, MA, USA). These analyses were performed to determine whether synthetic curcuminoids No. 11 and No. 12 could absorb blue light and function as PSs, similar to natural curcumin.

### 4.4. Extinction Coefficients

Stock solutions of the synthetic curcuminoids No. 11 and No. 12 photosensitizers and their reference counterparts were prepared in spectroscopic grade dimethyl sulfoxide (DMSO) at a concentration of 1.0 × 10^−3^ M. A series of working solutions with gradient concentrations ranging from 2.0 × 10^−6^ M to 2.0 × 10^−5^ were subsequently prepared by serial dilution to ensure that the maximum absorbance (*A*) did not exceed 1.0, thereby maintaining compliance with the linear range of the Beer–Lambert law. The absorption spectra were scanned in the wavelength range of 300–700 nm using a standard quartz cuvette with a 1.0 cm optical path length (*l*), with pure solvent utilized for baseline correction. To determine the molar extinction coefficient at the targeted blue-light wavelengths (e.g., 420 nm and 450 nm) as well as at the maximum absorption wavelength (λmax), the measured absorbance (*A*) was plotted against the corresponding molar concentration (*c*). The *ε* values were derived directly from the slope of the linear regression curve (*A = ε x c x l*), with a correlation coefficient (*R*^2^) greater than 0.999 deemed acceptable.

### 4.5. The Blue Light Photo-Irradiation System for Photodynamic Therapy

The photo-irradiation system for this study was according to our previous study [28]. The blue light intensity was 3.0 mW/cm^2^ using a DC 5V power supply. The LED (Vetalux Company, Tainan, Taiwan) emission spectra were from 410 to 510 nm with _max = 462 nm. These parameters of blue ligh photodynamic therapy were 3 mW/cm^2^ for 60 s, resulting in a total fluence of 0.18 J/cm^2^. The light dose of 0.18 J/cm^2^ was selected based on our preliminary optimization experiments, which demonstrated effective photoactivation of the curcuminoid analogs while minimizing phototoxicity caused by light irradiation alone. This relatively low fluence also allows clearer evaluation of compound-mediated photodynamic effects rather than nonspecific light-induced cellular damage [28].

### 4.6. Cytotoxicity and Cell Proliferation Studies (MTT Assays)

According to the method described by Kumar et al. [51], HaCaT cells were seeded into 96-well plates at a density of 4 × 10^3^ cells per well. After 24 h of incubation, the culture medium with DMSO was removed and replaced with either fresh medium alone (control group with DMSO) or experimental treatments. Experimental conditions included treatment with synthetic curcuminoids No. 11 or No. 12 at concentrations ranging from 0.1–10 µg/mL, 100 µM imiquimod (IMQ), or tert-butyl hydroperoxide (t-BuOOH) at concentrations of 200–800 µM.

For the curcuminoid treatment groups, cells were further divided into no-light and blue-light irradiation groups. Blue-light exposure was performed for 1 min using a light-emitting diode device (VetaLED Company, Taipei, Taiwan) with a maximum emission at 462 nm, a wavelength range of 410–510 nm, an irradiance of 3.0 mW/cm^2^, and a total light dose of 0.18 J/cm^2^. During irradiation, the culture plates were positioned approximately 1 cm from the light source. Following treatment, plates containing curcuminoid-treated cells were wrapped in aluminum foil to prevent additional light exposure and incubated for 24–72 h. In contrast, IMQ- and t-BuOOH-treated groups were incubated without aluminum foil for 0–8 h and 24 h, respectively.

After the designated incubation periods, the culture medium was removed, and 100 µL of MTT solution (0.5 mg/mL; 3-(4,5-dimethylthiazol-2-yl)-2,5-diphenyltetrazolium bromide) was added to each well. Cells were incubated at 37 °C for an additional 4 h to allow formation of purple formazan crystals. Subsequently, the supernatant was carefully aspirated, and 100 µL DMSO was added to dissolve the formazan product. Absorbance at 570 nm was measured using a spectrophotometer (Thermo Fisher Scientific, Waltham, MA, USA).

The MTT assay is a widely used quantitative colorimetric method for evaluating cell viability, proliferation, and cytotoxicity. Metabolically active cells reduce the water-soluble MTT reagent into insoluble purple formazan crystals, whereas damaged or nonviable cells lose this metabolic capability. Therefore, cell viability following treatment with synthetic curcuminoids was assessed by measuring MTT reduction. Using this assay, the maximal lower-cytotoxic concentrations of synthetic curcuminoids No. 11 and No. 12 in HaCaT cells were determined. The percentage of lower cytotoxicity in HaCaT cells was calculated as follows:

$$\mathrm{Cell}\;\mathrm{lower}\text{-}\mathrm{cytotoxicity}\;(\%)\;=\frac{OD570s}{OD570vc}\;\times 100\%$$

*OD570s*: Absorption value of experimental groups at 570 nm wavelength.

*OD570vc*: Absorption value of control groups at 570 nm wavelength.

### 4.7. Intracellular Reactive Oxygen Species (ROS) Analysis

According to the OxiSelectTM intracellular ROS assay kit (Cell Biolabs, Inc., San Diego, CA, USA), the fluorescent probe dichlorodihydrofluorescein diacetate (DCFH-DA) was used to detect intracellular ROS, which are primarily produced in the mitochondria of cells. DCFH-DA can enter cells upon addition and is then oxidized by intracellular ROS to highly fluorescent dichlorofluorescein (DCF). First, 10^4^ HaCaT cells/well were seeded in a dark 96-well plate and incubated for 24 h. Subsequently, the culture supernatant was carefully aspirated from each well, and the cells were washed three times with 100 µL/well of Hank’s Balanced Salt Solution (HBSS; 8 g/L NaCl, 0.4 g/L KCl, 1 g/L glucose, 60 mg/L KH_2_PO_4_, 47.5 mg/L Na_2_HPO_4_, pH 7.2). After adding 100 µL/well of 1× DCFH-DA medium and incubating for 1 h, the supernatant was aspirated, and each well was washed three times with HBSS. Following the addition of various concentrations of synthetic curcuminoids No. 11 or No. 12 for 1 h, except for the negative control (100 µL of medium), the remaining wells were treated with 100 µL/well of 200, 400, or 800 µM t-BuOOH and incubated at 37 °C for 1.5 h. t-BuOOH was used as an oxidant, inducing oxidative stress and thereby increasing ROS production within cells [52]. At this stage, intracellular DCFH-DA is converted to highly fluorescent DCF in the presence of ROS under blue light irradiation. Immediately, the fluorescence (Ex/Em = 480 nm/530 nm) of the samples was measured using a fluorescence spectrophotometer (Thermo Fisher Scientific). The percentage of the final product (highly fluorescent DCF) represents intracellular ROS production. This assay demonstrated that ROS generation in HaCaT cells treated with various concentrations of synthetic curcuminoids No. 11 or No. 12 increased in a dose-dependent manner under blue light irradiation. The calculations are as follows:

$$\mathrm{DCF}\;(\mathrm{ROS})\;\%\;=\frac{FLs}{FLvc}\;\times 100\%$$

*FLs*: Fluorescence intensity of experimental groups at 530 nm wavelength.

*FLvc*: Fluorescence intensity of negative control at 530 nm wavelength.

We designed distinct experimental groups, including a negative control without t-BuOOH, a group treated with t-BuOOH alone, a group treated with both t-BuOOH and quercetin (as a positive antioxidant control), and groups with curcuminoids No. 11 or No. 12 subjected to blue-light photodynamic irradiation. This setup enables us to understand how blue-light photodynamic irradiation enhances ROS production.

### 4.8. Enzyme-Linked Immunosorbent Assays (ELISA)

To assess cytokine production in HaCaT cells following treatment with synthetic curcuminoids No. 11 or No. 12, cells at approximately 80–90% confluence were used as fully differentiated cultures. Cells were co-treated with 5 µg/mL No. 11 or 1 µg/mL No. 12, corresponding to their maximal non-cytotoxic concentrations as determined by the MTT assay (Figure 2), together with 100 µM IMQ for 1 h. After treatment, culture supernatants were collected by centrifugation at 300× *g* (1500 rpm), aliquoted, and stored at −80 °C until analysis. The concentrations of IL-17 and IL-8 in the collected supernatants were quantified using ELISA kits (Thermo Fisher Scientific) according to the manufacturer’s instructions. Cytokine levels were compared between cells treated with IMQ alone and those co-treated with IMQ and synthetic curcuminoids No. 11 or No. 12.

### 4.9. Real-Time qRT-PCR

Quantitative real-time PCR (qRT-PCR) was performed to evaluate the mRNA expression levels of IL-17A, IL-17F, IL-8, ERK1, and ERK2 in HaCaT cells following treatment with synthetic curcuminoids No. 11, No. 12, and/or IMQ. Briefly, HaCaT cells at 80–90% confluence were treated with 5 µg/mL curcuminoid No. 11 or 1 µg/mL curcuminoid No. 12 in the presence of 100 µM IMQ for 1 h.

After treatment, cells were harvested using trypsin–EDTA, and total RNA was extracted according to the manufacturer’s protocol (Thermo Fisher Scientific). The isolated RNA was then used for cDNA synthesis, and gene expression analysis was conducted using a qPCR kit (Promega, Madison, WI, USA).

For each reaction, a total volume of 20 µL was prepared, containing 10 µL qPCR master mix, 4 µL cDNA template, 1 µL forward primer, 1 µL reverse primer, 0.2 µL CXR reference dye, and nuclease-free water to bring the final volume to 20 µL. Amplification was performed using a real-time PCR system (Roche, Basel, Switzerland) under the following cycling conditions: initial pre-incubation at 95 °C for 2 min, followed by 45 cycles of denaturation at 95 °C for 15 s, annealing at 60 °C for 1 min, and extension at 72 °C for 10 s.

Melting curve analysis was subsequently performed to confirm product specificity, starting at 95 °C for 5 s, then 65 °C for 1 min, and gradually increasing to 97 °C. The run was completed with a cooling step to 40 °C. Primer sequences used for qPCR amplification are listed in Table 1.

### 4.10. Western Blotting Assays

According to the standard protocol described by Burnette [30], protein samples were mixed with 3× sodium dodecyl sulfate polyacrylamide gel electrophoresis (SDS-PAGE) sample buffer (12 mL glycerol, 6 mL 20% SDS, 3 mL 1 M Tris pH 6.8, 3 mL β-mercaptoethanol, 0.006 g bromophenol blue) in a 2:1 ratio and boiled for 5 min. After cooling on ice for 5 min, the protein samples were loaded onto 10% SDS-PAGE gels and electrophoresed at 150 V to separate proteins based on their molecular weights. Subsequently, the proteins were transferred onto a polyvinylidene difluoride membrane at 400 mA. The membrane was then treated with blocking buffer (5% skim milk, 20 µM Tris, 0.15 µM NaCl, 0.1% Tween-20) for 1 h to prevent non-specific protein binding to the membrane. The primary antibody (diluted in blocking buffer) was added to the membrane and incubated overnight at 4 °C with shaking. After washing with 20% TBST buffer/L (400 mL 1 M Tris pH 7.5, 20 mL Tween-20, 175.3 g NaCl pH 7.5) three times for 10 min each, the secondary antibody (diluted in blocking buffer) was added and incubated for 1 h with shaking. Following three additional washes with TBST, a chemiluminescent substrate (Thermo Fisher Scientific) was added and incubated for 5 min. The membrane was then imaged using a chemiluminescence detector (Thermo Fisher Scientific). ImageJ v1.54t (National Institute of Health) was used to determine the relative quantities of the detected proteins.

### 4.11. Statistical Analysis

The experiments were performed in at least triplicate, and the data are expressed as mean ± standard deviation of individual experiments. The data were assessed by analysis of variance (one-way ANOVA) and *t*-test using SPSS Statistics v32 (IBM, Armonk, NY, USA). * *p* < 0.05; ** *p* < 0.01; **** *p* < 0.0001 were considered significant.

## 5. Conclusions

Synthetic curcuminoids No. 11 and No. 12 demonstrated multiple favorable characteristics for PDT applications, including strong blue-light absorption, efficient intracellular ROS generation, anti-inflammatory activity, minimal cytotoxicity, and the ability to promote apoptosis in psoriasis-like keratinocytes. These findings indicate that combining synthetic curcuminoids with blue-light-mediated PDT may provide a promising anti-inflammatory potential for psoriasis. Owing to their potential safety, cost-effectiveness, and suitability for topical administration, synthetic curcuminoids No. 11 and No. 12 may serve as valuable candidates for the development of next-generation PDT agents targeting inflammatory skin disorders, particularly psoriasis.

## Figures and Tables

**Table 1:** Primer sequences.

Genes	Primer Sequences (5′-3′)	Design Sources
*IL-17A*	F: TCTGTGATCTGGGAGGCAAAG R: CGTTCCCATCAGCGTTGAT	[39]
*IL-17F*	F: GCTGTCGATATTGGGGCTTG R: GGAAACGCGCTGGTTTTCAT	[40]
*IL-8*	F: CTGGCCGTGGCTCTCTTG R: CCTTGGCAAAACTGCACCTT	[41]
*ERK1*	F:TGGCAAGCACTACCTGGATCAG R:GCAGAGACTGTAGGTAGTTTCGG	Origene™ Technologies (Rockville, ML, USA)
*ERK2*	F: ACACCAACCTCTCGTACATCGG R:TGGCAGTAGGTCTGGTGCTCAA	Origene™ Technologies (Rockville, ML, USA)

## Data Availability

The data presented in this study are available on request from the corresponding author.

## Abbreviations

The following abbreviations are used in this manuscript:

PDT	photodynamic therapy
UV-Vis	ultraviolet–visible
ROS	reactive oxygen species
IMQ	Imiquimod
CXCL1	chemokine (C-X-C motif) ligand 1
IL-8	interleukin-8
IL-17	interleukin-17
TA	transit-amplifying
PS	photosensitizer
ELISA	enzyme-linked immunosorbent assay
qRT-PCR	Quantitative real-time PCR
DCF	dichlorofluorescein
DMSO	dimethyl sulfoxide
DCFH-DA	dichlorodihydrofluorescein diacetate
t-BuOOH	tert-butyl hydroperoxide
HBSS Hank’s	Balanced Salt Solution
SDS-PAGE	sodium dodecyl sulfate polyacrylamide gel electrophoresis
ERK	extracellular signal-regulated kinase
NF-κB	nuclear factor-kappa B
MAPK	mitogen-activated protein kinase
5-ALA	5-aminolevulinic acid
MAL	methyl aminolevulinate
